# Antifungal, toxicological, and colorimetric properties of *Origanum vulgare*, *Moringa oleifera*, and *Cinnamomum verum* essential oils mixture against Egyptian Prince Yusuf Palace deteriorative fungi

**DOI:** 10.1186/s12896-024-00940-8

**Published:** 2025-01-07

**Authors:** Asmaa Alhussein Mohamed, Mahgoub A. Ahmed, Abdallah S. Korayem, Samah H. Abu-Hussien, Wael Bakry Rashidy

**Affiliations:** 1 Faculty of Archaeology, South Valley University, Qena, Egypt; 2 Department of Agricultural Microbiology, Faculty of Agriculture, Ain Shams University, Cairo, 11241 Egypt

**Keywords:** Essential oils, Fungal biodeterioration, Wall paintings, Conservations, Antimicrobial

## Abstract

The increasing demand for sustainable alternatives to conventional antifungal agents has prompted extensive research into the antifungal properties of plant essential oils (EOs). This study investigates the use of EOs mixture (*Origanum vulgare*, *Moringa oleifera*, and *Cinnamomum verum*) for controlling fungal deterioration in wall paintings at the archaeological Youssef Kamal Palace in Nag Hammadi, Egypt. Fungal isolates were collected from deteriorated wall paintings and identified using phenotypic and genotypic analyses. *Aspergillus sp.* was found to be the predominant species (50%), followed by *Penicillium* sp. (16.7%), *Fusarium* sp. (16.7%), and others. They were genetically identified to be *Aspergillus oryzae*, *Aspergillus niger*, *Penicillium chrysogenum*, *Fusarium solani*, *Alternaria alternata*, *Botrytis cinerea*, and *Trichoderma viride*. The antifungal activity of three individual oils (oregano, moringa and cinnamon) was evaluated against the most predominant *A. niger* strain. Out of the three oils, oregano oil showed the strongest antifungal effect with an inhibition zone diameter (IZD) of 4.5 cm followed by moringa (3.5 cm) and cinnamon (3.2 cm). A mixture design approach optimized the EOs combination, with the most effective composition being (44% oregano, 46% moringa, 10% cinnamon), yielding an IZD of 6.5 cm. The optimized EOs mixture demonstrated complete inhibition against all tested fungal strains. The minimal inhibitory concentration tests showed varying efficacies against different fungal strains, with MIC values ranging from 125 to 500 µg/mL. GC-MS analysis identified the major bioactive compounds: carvacrol (83.25%) in oregano, trans-13-octadecenoic acid (22.62%) in moringa, and cinnamaldehyde (24.42%) in cinnamon. Cytotoxicity testing on human skin fibroblasts (HSF) showed minimal toxicity of EOs mixture with 87.64% cell viability at 100 µg/ml. Colorimetric measurements revealed some colour changes in experimental painting samples, particularly with cinnamon oil on white pigment (ΔE = 9.64) and moringa oil on a yellow pigment (ΔE = 16.31). However, oregano oil consistently showed the least impact across all pigments. These findings demonstrate the potential of the EOs combination as an effective, eco-friendly approach to mitigating fungal deterioration in wall paintings, contributing to sustainable conservation strategies for cultural heritage preservation.

## Introduction

Wall paintings and architectural surfaces in historical buildings represent invaluable cultural heritage, providing insights into past civilizations’ artistic expressions, social structures, and technological achievements. However, these artifacts are continuously threatened by various environmental factors, with microbial deterioration being one of the most significant challenges [1]. The proliferation of microorganisms, particularly fungi, on these surfaces can lead to aesthetic damage, structural weakening, and even irreversible loss of historical and artistic value. The issue of biodeterioration in cultural heritage sites has been extensively studied over the past few decades [2]. Researchers have identified numerous fungal species responsible for the degradation of wall paintings and other architectural surfaces. The fungi belonging to genera *Aspergillus* sp., *Penicillium* sp., *Cladosporium* sp., *Fusarium* sp., *Trichoderma* sp., *Botrytis* sp., and *Alternaria* sp. are commonly found on deteriorated wall paintings across various climatic regions. These microorganisms can cause significant damage through various mechanisms, including the production of organic acids, pigments, and enzymes that can alter or degrade the substrate materials [3].

The historical Youssef Kamal Palace in Nag Hammadi, Egypt serves as a prime example of a cultural heritage site facing the challenges of microbial deterioration. It was built during the late 19th century, this architectural masterpiece showcases a unique blend of Islamic, Ottoman, and European design elements. However, like many historical buildings, it is susceptible to biodeterioration, particularly in its ornate wall paintings and decorative elements. Traditional approaches to mitigating microbial growth on cultural heritage artifacts have often relied on chemical biocides. However, these treatments can sometimes cause additional damage to the artifacts and pose health risks to conservators and visitors [4]. Furthermore, the overuse of conventional antimicrobial agents has led to concerns about the development of resistant microbial strains [5]. These issues have prompted researchers to explore alternative, more sustainable approaches to conservation.

In recent years, there has been growing interest in the use of natural products, particularly essential oils, as potential antimicrobial agents in the field of cultural heritage preservation. Essential oils derived from plants have been used for centuries for their medicinal and antimicrobial properties. Their complex composition of various bioactive compounds often results in broad-spectrum antimicrobial activity against bacteria, fungi, and even viruses [6]. Several studies have investigated the efficacy of essential oils against microbial strains commonly associated with the biodeterioration of cultural heritage artefacts. For example, Mahidi et al. [7] demonstrated the antifungal activity of clove and cinnamon essential oils against fungi isolated from cultural heritage objects in Serbia. Similarly, the effectiveness of various essential oils, including tea tree and thyme, against fungal strains isolated from documents was reported [8]. The potential of essential oils as antimicrobial agents in cultural heritage conservation extends beyond their direct biocidal effects. Many essential oils also possess antioxidant properties, which could potentially help to protect artifacts from oxidative damage [9]. Additionally, some essential oils have been shown to have water-repellent characteristics, which could provide an added layer of protection against moisture-related deterioration [10].

However, the application of essential oils in cultural heritage conservation is not without challenges. The volatile nature of many essential oil components can lead to rapid evaporation, potentially limiting their long-term effectiveness [11]. There are also concerns about the potential for some essential oils to cause discoloration or other forms of damage to sensitive materials [12]. Therefore, careful selection and thorough testing of essential oils are crucial before their application in conservation treatments.

The use of plant essential oils (EOs) as antimicrobial agents in cultural heritage conservation aligns well with the growing emphasis on green and sustainable conservation practices. Unlike synthetic biocides, EOs are biodegradable and generally considered environmentally friendly [13]. This aspect is particularly important in the context of cultural heritage sites, where the long-term environmental impact of conservation treatments must be carefully considered.

Recent advancements in analytical techniques have allowed for more detailed characterization of the chemical composition of essential oils and their mechanisms of action against various microbial species. Gas chromatography-mass spectrometry (GC-MS) analysis has been widely used to identify the bioactive compounds in essential oils responsible for their antimicrobial properties [14]. This information is crucial for optimizing the selection and application of essential oils in conservation treatments.

The antimicrobial effects of combining different essential oils have also been a subject of recent research. Some studies have shown that certain combinations of essential oils can exhibit enhanced antimicrobial activity compared to individual oils [15]. This approach could potentially allow for the development of more effective and broad-spectrum treatments for microbial control in cultural heritage conservation. While the antimicrobial properties of essential oils are well-documented, their potential effects on the physical and chemical properties of the materials they are applied to must also be carefully evaluated. This is particularly important in the context of cultural heritage conservation, where maintaining the integrity and authenticity of the artifacts is paramount. Some researchers have begun to investigate the impact of essential oil treatments on various substrates commonly found in cultural heritage objects, such as stone, wood, and paper [10].

The application methods for essential oil treatments in cultural heritage conservation also warrant careful consideration. Traditional methods such as spraying or brushing may not always be suitable, particularly for fragile or highly valuable artifacts. Some researchers have explored innovative approaches, such as the use of nanotechnology to encapsulate essential oils, potentially allowing for more controlled and sustained release of the active compounds [7, 15].

In the specific context of wall paintings, the interaction between essential oils and the complex, multi-layered structure of these artifacts presents unique challenges and opportunities. The porosity and chemical composition of the paint layers and underlying plaster can significantly influence the penetration and efficacy of essential oil treatments. Some studies have suggested that the incorporation of essential oils into consolidation treatments could potentially provide dual benefits of structural reinforcement and microbial control [15]. The aim of this study was to investigate the potential of a combination of plant essential oils (oregano, moringa, and cinnamon) as an eco-friendly alternative for controlling fungal deterioration in the wall paintings of the historical Youssef Kamal Palace in Nag Hammadi, Egypt. We seek to identify the predominant fungal species responsible for biodeterioration at the site, evaluate the antimicrobial efficacy of the selected essential oil mixture against these isolates, and assess the potential impacts of the treatment on the aesthetic and physical properties of the wall paintings. Additionally, the study aims to optimize the composition of the essential oil mixture for maximum effectiveness while minimizing any potential adverse effects on cultural heritage materials.

## Materials and methods

### Site and sample collection

**Fig. 1:**
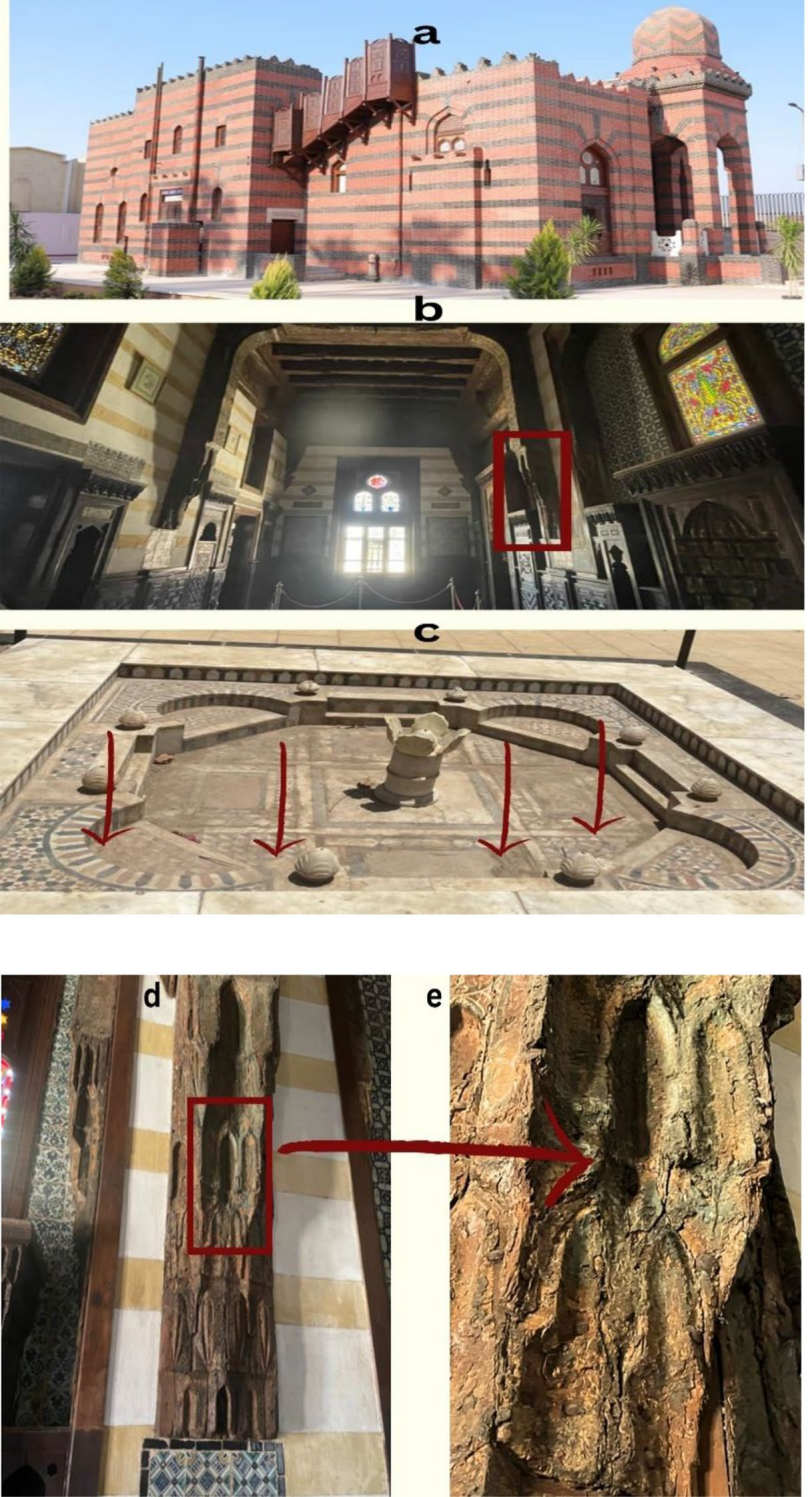
Collection sites from Prince Youssef Palace located in Nag Hammadi, Qena, Egypt. (**a**): Prince Youssef Kamal Palace in the city of Nag Hammadi, north of Qena Governorate. (**b**): General view of the southern hall of Prince Yusuf Kamal Palace. (**c**): Sampling locations at the marble fountain in the courtyard of Prince Yusuf Kamal Palace. (**d**): The wooden stalactite in the southern hall of Prince Yusuf Kamal Palace, where microbial samples were collected. (**e**): A detailed picture of the location where microbial samples were collected from the wooden stalactite in the southern hall of Prince Yusuf Kamal Palace

Yusuf Kamal Palace, located in Nag Hammadi, Qena, Egypt, is a historical mansion that stands as an architectural masterpiece of the late 19th century (Fig. 1). It was built during the era of Khedive Abbas II, who ruled Egypt from 1892 to 1914 and was designed by the renowned French architect Emmanuel Brune [16]. The palace features a unique blend of architectural styles, incorporating Islamic, Ottoman, and European design elements. Its ornate façade, intricate carvings, and beautifully landscaped gardens characterize the structure. Notably, the palace’s main entrance is an exquisite example of the fusion of these styles, with Islamic arches and European-style columns. The interior of Prince Youssef Kamal Palace is equally impressive, boasting elaborately decorated rooms with exquisite frescoes, stucco work, and intricate woodwork. The palace’s architectural features and design reflect the opulence and grandeur of the period during which it was constructed [17].

For samples collection, sixty sterile cotton swabs were utilized to collect samples from the deteriorated wall paintings and the marble fountain in the courtyard exhibiting significant damage at Prince Youssef Kamal Palace in Nag Hammadi, Qena, Egypt (Fig. 1). Subsequently, the swabs were placed into sterile tubes and conveyed to the Microbiology Laboratory at the Faculty of Agriculture, Ain Shams University, Egypt.

### Plant essential oils

All used essential oils; *Origanum vulgare*, *Moringa oleifera*, and *Cinnamomum verum* were obtained from National Research Center, Cairo, Egypt.

### Isolation of deteriorative fungi

To isolate fungi, plates of potato dextrose agar (PDA) medium [18] were prepared and streaked with cotton swabs samples. Subsequently, all plates were incubated for 3–5 days at 25 °C. Following incubation, all colonies were picked up and subjected to purification through successive subcultures on the surface of PDA medium. All purified isolates were then stored at 4 °C for further investigations [19].

### Fungal phenotypic identification

The fungal isolates showed different cultural characteristics were identified according to their cell morphological and cultural properties using slide culture technique [20].

### Fungal genotypic identification

The fungal colonies were sent to the Molecular Biology Research Unit at Assiut University for molecular identification through 18S rRNA sequencing and DNA extraction using a Patho-gene-spin DNA/RNA extraction kit from Intron Biotechnology Company, Korea. Subsequently, fungal DNA samples were forwarded to SolGent Company in Daejeon, South Korea, for PCR and rRNA gene sequencing. PCR of the chosen isolates was conducted using ITS1 (forward) and ITS4 (reverse) primers: ITS1: (5′-TCC GTA GGT GAA CCT GCG G-3′) and ITS4: (5′-TCC TCC GCT TAT TGA TAT GC-3′). The reaction mixture included ddNTPs, and the resulting purified recombinant product was sequenced using the respective primers. The phylogenetic trees exhibited here were built together by employing the neighbor-joining algorithm using MEGA (Molecular Evolutionary Genetics Analysis) software, version 11 with bootstrap analysis conducted with 1000 replicates to assess the reliability of the tree topology [19].

### Antifungal activities of essential oils against predominant isolate

For antifungal activity, the three essential oils (oregano, moringa and cinnamon) were tested using the agar well diffusion method. Briefly, the most predominant fungal strain was cultivated on PDA slants at 25 °C for 5 days. Spores were harvested using 5mL sterile phosphate-buffered solution (pH 7.2) supplemented with 0.05% (v/v) Tween 80 (Merck, Darmstadt, Germany). The spores were gently loosened using a sterile inoculating loop. 50 µl of the most predominant fungal strain spores suspension (10^6^ CFU/mL) were streaked on the surface of Mueller Hinton Agar [18].

Using a 6 mm diameter cork porer, wells were made in 15 cm agar plates and inoculated with 100µL of the three EOs. Well with 100 µL of sterile distilled water was used as a control. Plates were then incubated at 25 °C for 3–5 days. After the incubation period, the Inhibition zones diameters (IZD)in cm were measured [21]. All experiments were carried out in triplicates.

### Mixture design for the optimization of EOs mixture against the most predominant fungal strain

The mixture design of RSM was used to calculate all mixture constitutions levels against the most predominant fungal strain, growth inhibition was expressed as IZD measured in cm. The Sum of all mixture constitutes was expressed mathematically as the following;

- 0.000 ≤ A: Oregano ≤ 0.660,
- 0.000 ≤ B: Moringa ≤ 0.660,
- 0.000 ≤ C: Cxinnamon ≤ 0.660,

A + B + C = 1.000, in which this relationship is called the fundamental constraint of mixtures. An optimal design with a full experiment of 16 runs was chosen to be used in the optimization process as presented in Table 1. The 3D triangle was designed to have the three diluted essential oils located in the mixtures of essential oils, the equal portions mixture of the three components at the vertices of the triangle. Run trial No. 5 and 6 were replicated 2 times to detect the pure error and to compare it with the lack of fit. Cubic and quadratic models were used to express responses as a function of independent variables based on the mixture design method in which Y represents the response (inhibition zone diameter in cm). α1, α2, α3 represent the linear term coefficients. α12, α22, and α23 represent the binary term coefficients. α123 was for the ternary term coefficient [5].

**Table 1:** Matrix for the mixture design of plant essential oils combination

Run	Essential oil (mL)			
	Oregano	Moringa	Cinnamon	
1	0.44	0.46	0.10	
2	0.47	0.00	0.53	
3	0.66	0.34	0.00	
4	0.26	0.07	0.67	
5	0.26	0.66	0.08	
6	0.00	0.50	0.50	
7	0.26	0.66	0.08	
8	0.35	0.32	0.33	
9	0.56	0.22	0.22	
10	0.66	0.00	0.34	
11	0.35	0.32	0.33	
12	0.35	0.32	0.33	
13	0.00	0.50	0.50	
14	0.05	0.66	0.29	
15	0.07	0.27	0.66	
16	0.35	0.32	0.33	

### The minimum inhibitory concentration (MIC) of the optimum EOs mixture against fungal strains

The minimum inhibitory concentration of the optimum EOs mixture determined by RSM against each identified fungal strains was investigated using the agar well diffusion method [22]. Briefly, a stock solution of EOs mixture of 1000 µg/mL was prepared using 10% dimethyl sulfoxide then two fold serial dilutions of 1000, 500, 250, 125, and 62.5 µg/mL were prepared. 1 ml of each fungal spore suspension (10^6^CFU/mL) was pipetted into sterile Petri dishes followed by the addition of molten PDA agar then mixed well. Using 6 mm diameter cork porer, wells were made in the seeded agar plates and 100 µL of each EOs mixture concentration was transferred to the respective well. Plates were kept in the refrigerator for 30 min for diffusion then incubated at 25 °C for 3–5 days. Fluconazole as commercial standard antibiotic at the same concentrations was used as positive control. After incubation, the Inhibition zone diameter (cm) was measured. The MIC is defined as the lowest concentration of the oils mixture that produces a visible zone of inhibition. All trials were carried out in triplicates.

### Gas-mass chromatography (GC/MS) analysis of EOs

The chemical composition of the three used essential oils was analyzed using a Trace GC1310-ISQ mass spectrometer (Thermo Scientific, Austin, TX, USA) equipped with a direct capillary column TG-5MS (30 m × 0.25 mm × 0.25 μm film thickness). The comprehensive analytical method employed a programmed temperature gradient: the column oven was initially maintained at 50 °C, then incrementally increased by 5 °C/min to 230 °C (held for 2 min), subsequently raised to a final temperature of 290 °C at a rate of 30 °C/min (held for 2 min). The injector and MS transfer line temperatures were precisely controlled at 250 °C and 260 °C, respectively, with helium serving as the carrier gas at a constant flow rate of 1 ml/min. Sample preparation involved automatic injection of 1 µl diluted samples using an autosampler AS1300 coupled with the gas chromatograph in split mode. Electron ionization (EI) mass spectra were acquired at 70 eV ionization voltages, scanning the mass range of m/z 40–1000 in full scan mode, with the ion source temperature maintained at 200 °C. Compound identification was performed by comparing the obtained retention times and mass spectra against the comprehensive WILEY 09 and NIST 11 mass spectral databases, ensuring accurate and reliable chemical characterization [23, 24].

### Cytotoxicity of the optimum EOs mixture against normal human skin fibroblast (HSF)

Human skin fibroblast (HSF) cells acquired from Nawah Scientific Inc. (https://nawah-scientific.com) were cultivated in Dulbecco’s Modified Eagle’s Medium (DMEM) supplemented with 10% fetal bovine serum, 100 mg/mL streptomycin and 100 U/mL penicillin at 37 °C in a 5% CO_2_ humidified atmosphere. The MTT assay was utilized to evaluate the cytotoxicity of the EOs mixture by quantifying HSF cell viability after treatment with different concentrations [25].

### Colorimetric measurements of EOs on experimental paintings

For colorimetric alterations, the National Institute of Standards (NIS) in Cairo, Egypt, utilized the Optimatch 3100^®^ from SDL Company to measure the colour changes caused by three plant essential oils (oregano, moringa and cinnamon) and the optimum mixture on experimental painting samples before and after treatment. The CIE L*a* b* system was employed to record the colour variations, with the L value representing brightness, the a* value representing red-green, and the b* value representing yellow-blue. The total colour changes (E) before and after treatment were calculated using the following equation [19].

$$\Delta {\rm E}\, = \,\sqrt {{{(\Delta L)}^2}\, + \,{{(\Delta a)}^2}\, + \,{{(\Delta b)}^2}}$$

where L (lightness), a (red/green axis), and b (yellow/blue axis) values were recorded.

### Statistical analysis

Data were analyzed by one-way ANOVA followed by Tukey’s posthoc test [26] using SPSS 12. *P* < 0.05 was considered statistically significant. The data for mixture design were statistically analyzed using Design Expert 13 Statistics software (https://www.statease.com/docs/v13/).

## Results

### Isolation of deteriorative fungi from Prince Youssef Palace

**Fig. 2:**
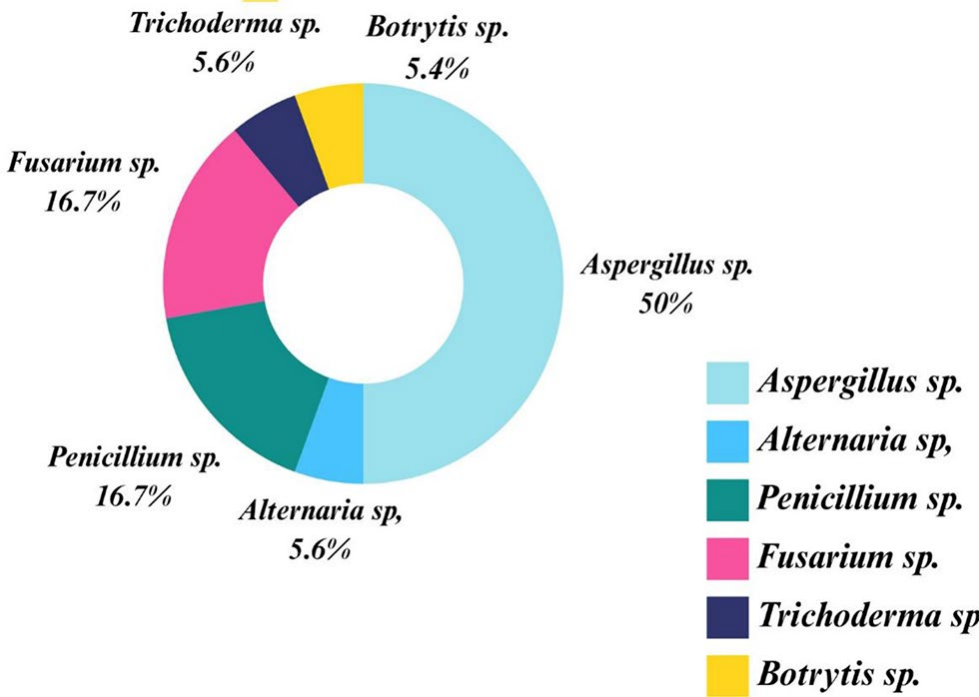
The relative abundance (%) of different fungal species identified from the collected samples from Prince Yusuf Palace

One Hundred Twenty-Five fungal isolates were obtained. Figure 2 presents the relative abundance of different fungal genera identified from the collected samples. The most dominant genus is *Aspergillus* sp., comprising 50% of the total detected fungi detected so, it was selected for further experiments. The isolated fungi of potential deteriorative activity were *Alternaria* sp. (5.6%), *Trichoderma* sp. (5.6%), *Botrytis* sp. (5.4%), *Fusarium* sp. (16.7%), and *Penicillium* sp. (16.7%), which account for a significant portion of the identified fungi. The presence of these deteriorative fungal genera is noteworthy, as species belonging to these genera are commonly associated with material spoilage and biodegradation in various environments.

### Fungal phenotypic identification

**Fig. 3:**
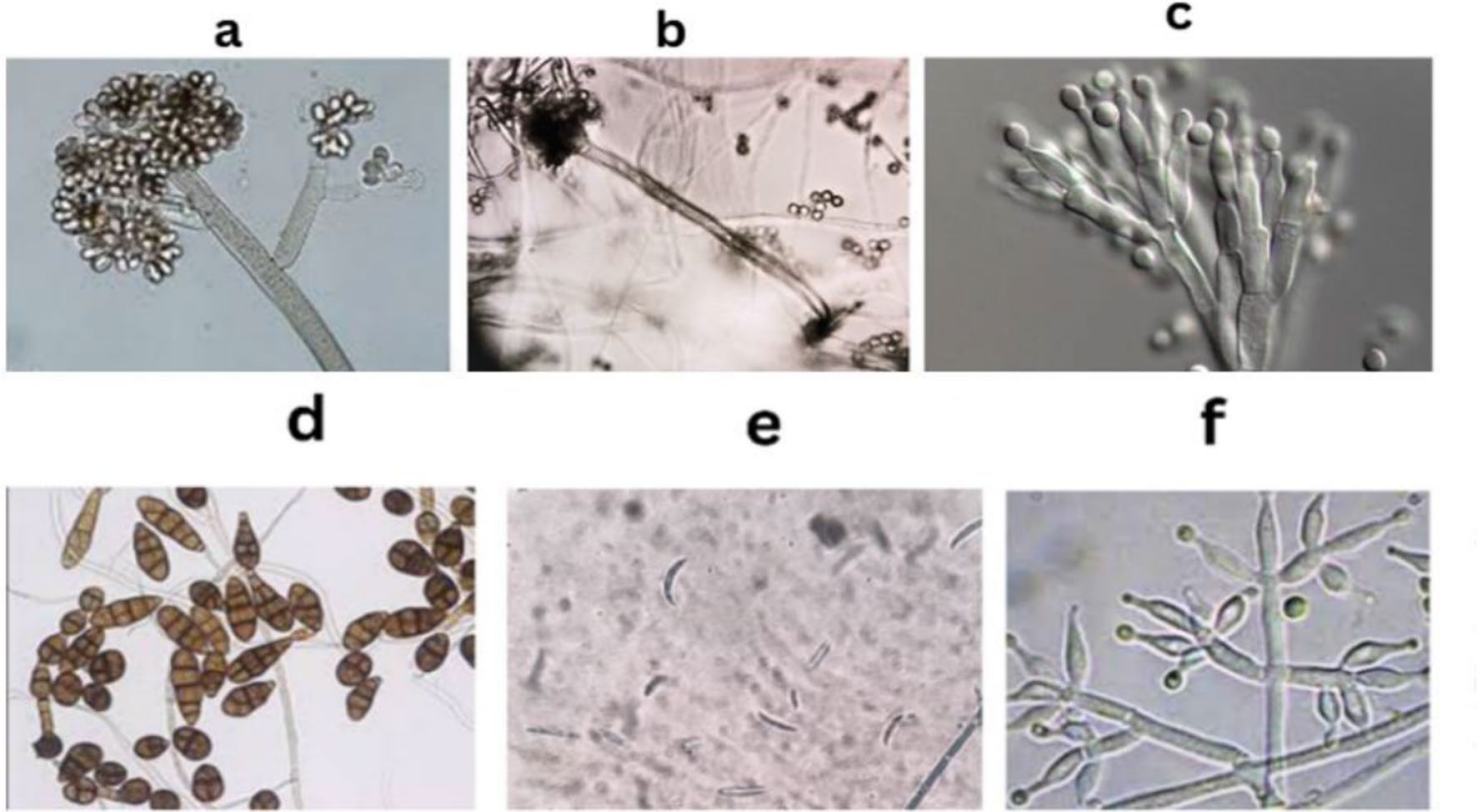
Diverse morphological features of the obtained fungal isolates from Prince Youssef Palace. (**a)**: *Botrytis* sp.(**b**): *Aspergillus* sp. (**c**): *Penicillium* sp. (**d**): *Alternaria* sp. (**e**): *Fusarium* sp. (**f**): *Trichoderma sp.* microscopic examinations were carried out using 100X magnification power

The fungal genera represented in Fig. 3 exhibit diverse morphological features. *Aspergillus* sp., the most predominant, likely displays septate hyphae with conidiophores bearing globose conidia arranged in chains. For *Botrytis* sp., The mycelium was hyaline to brown, septate, and branching and the conidia seemed gray in mass, yet they were solitary, hyaline, or pale brown. *Penicillium* sp. displays a brush-like conidiophore structure with chains of spherical conidia. *Alternaria* sp. and *Fusarium* sp. tend to have elongated, multi-celled conidia borne on distinctive conidiophores. *Trichoderma* sp. exhibits highly branched conidiophores with clusters of green-pigmented conidia.

### Molecular identification using 18 S rRNA gene analysis

**Fig. 4:**
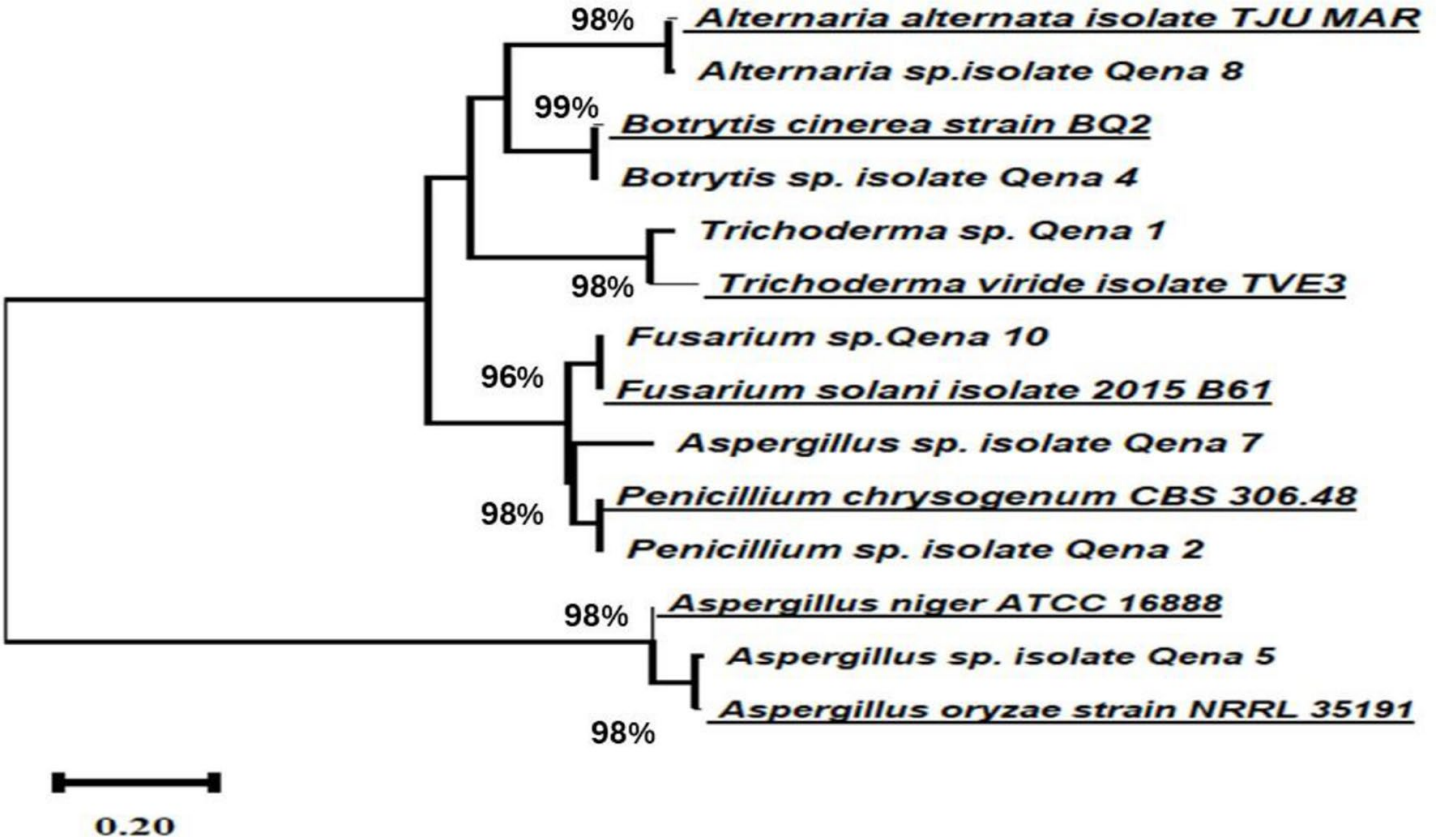
Phylogenetic analysis of the obtained fugal strains into the genetic diversity and relationships among the fungal isolates using Neighbor-joining tree based on 18 S rRNA sequences obtained from BLAST search indicating the position of each isolate and related strains

Fig. 4 presents the phylogenetic tree with various fungal isolates obtained from deteriorated archaeological wall paintings. The tree reveals the evolutionary relationships among the isolates obtained based on their genetic similarities. The isolates were deposited in GenBank, a publicly accessible database for nucleotide sequence data. *A. alternata* isolate TJU MAR and *Alternaria* sp. isolate Qena 8 (OQ726571.1) exhibited 98% similarity, indicating close relatedness. *B. cinerea* strain BQ2 and *Botrytis* sp. isolate Qena 4 (OQ726567.1) showed 99% similarity, suggesting a closer evolutionary tie. *Trichoderma* sp. Qena 1 (OQ726568.1) and *Trichoderma viride* isolate TVE3 displayed 98% similarity. *Fusarium* sp.Qena 10 (OQ726572.1) and *Fusarium solani* isolate 2015 B61 shared 96% similarity. *Aspergillus* sp. isolate Qena 7 (OQ726570) and *Penicillium chrysogenum* CBS 306.48 exhibited 98% similarity, indicating close relatedness. *Aspergillus niger* ATCC 16,888 and *Aspergillus* sp. isolate Qena 5 (OQ726568.1) shared 98% similarity, while *A. oryzae* strain NRRL 35,191 displayed 98% similarity.

### Antifungal activity of EOs against *A. niger*

**Fig. 5:**
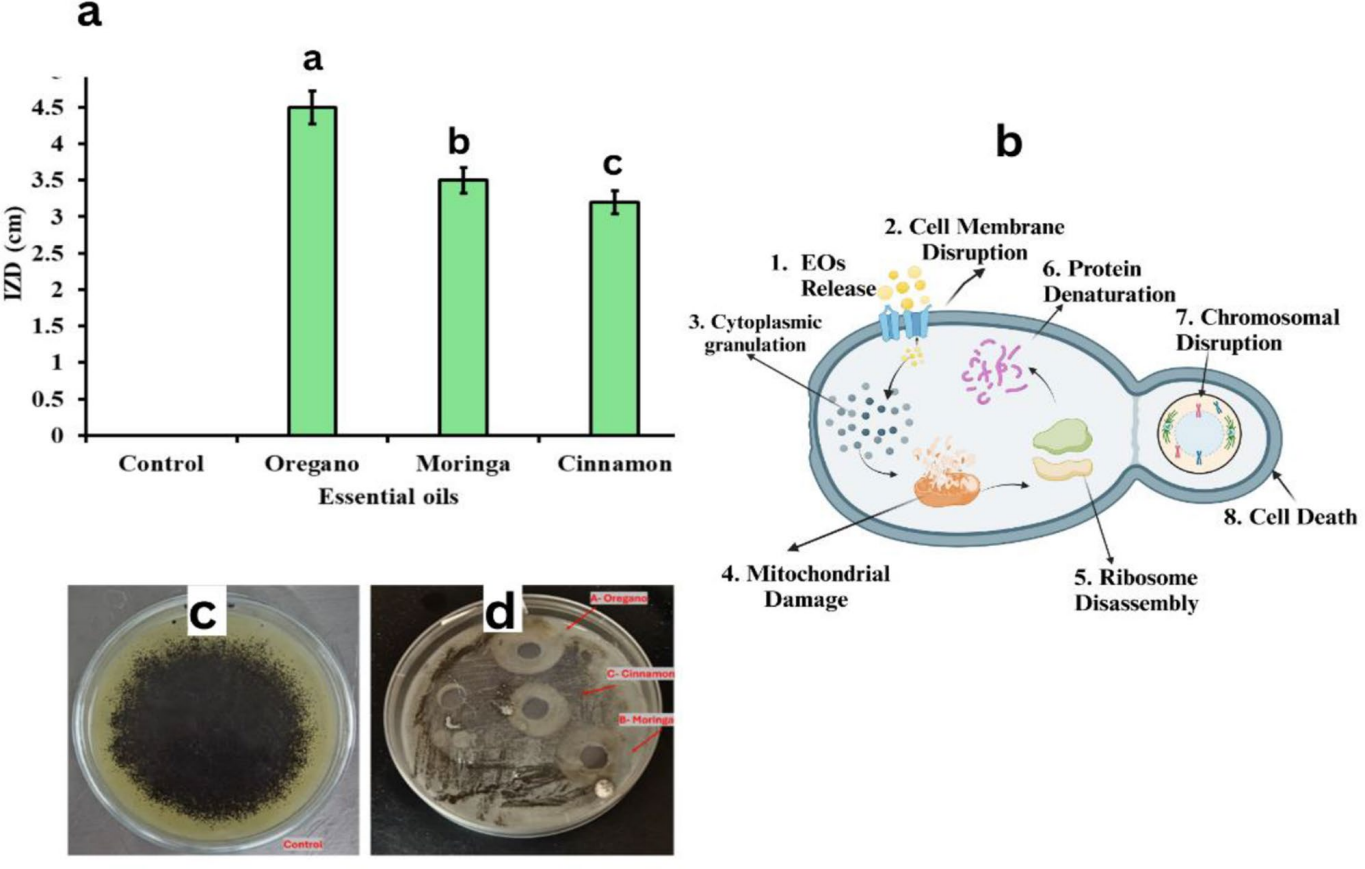
Antifungal activity of the selected EOs (oregano, moringa and cinnamon) against *A.niger* isolated from Prince Youssef Kamal Palace.(**a**): Inhibition zone diameters (cm) for the three oils. The bars represent the mean ± SD (*n* = 3). Significant differences are shown in different letters (*P* < 0.05). (**b**): Mechanism of action of oils against fungal cell. (**c**): Control of *A.niger* growth. (**d**): Effect of the three oils on *A.niger* growth using well diffusion method

The antifungal activity of different essential oils against *A. niger* as the most dominant strain was presented in Fig. 5. as measured by the inhibition zone diameter (IZD). The results show that the oregano essential oil had the highest antifungal activity, with an IZD of approximately 4.5 cm. This is followed by the moringa and cinnamon essential oils, which had IZDs of around 3.5 cm and 3.2 cm, respectively. The control well, which did not contain any essential oil, had an IZD of 0 cm.

### Antifungal activities of EOs mixture against *A. niger*

**Fig. 6:**
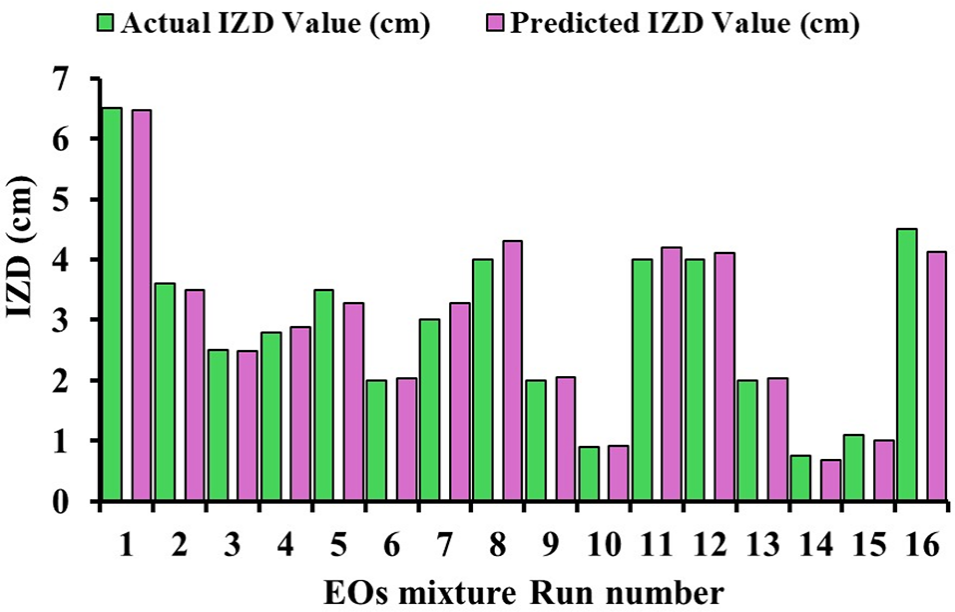
Experimental and predicted values for *A.niger* inhibition IZD (cm) using oregano, moringa, and cinnamon optimum mixtures

Figure 6 represents 16 experimental runs exploring the antifungal activities of a mixture of three plant essential oils (oregano, moringa and cinnamon) against the fungal strain *A. niger* (predominant strain) expressed as IZD (cm). Run 1 demonstrated the highest actual IZD value at 6.5 cm, closely matching its predicted value of 6.44 cm, with a balanced composition of 0.44 oregano, 0.46 moringa, and 0.1 cinnamon oils. In contrast, run 2 showed a significantly lower IZD of 2.5 cm (predicted 2.49 cm) with a composition heavily weighted towards oregano (0.47) and cinnamon (0.53), but zero moringa. Runs 8, 11, and 12 exhibited near-identical compositions (0.35 oregano, 0.32 moringa, 0.33 cinnamon) with IZD values of 4.3, 4.2, and 4.1, respectively. The lowest performance was observed in run 10, with an IZD of only 0.9 cm, and run 14 with 0.75 cm, despite different oil proportions. Notably, runs 7 and 5 showed moderate IZD values of 3 and 3.5 cm, with significantly different oil compositions.

ANOVA results were illustrated in Table 2 for the cubic model of the mixture design. The *F*-value of 100.83 implies a highly significant model (*P*-value < 0.0001), indicating the capability of model terms to explain the variation in the response variable (IZD). Significant model terms include linear mixture terms (A, B), interaction terms (AB, AC, BC, ABC), and quadratic terms (AB(A-B), AC(A-C)), except for term BC (B-C) (*P* = 0.0934). The Lack of Fit *F*-value of 0.47 is non-significant (*P* = 0.5218), suggesting a desirable model fit to the experimental data without a significant lack of fit. With an R² value of 0.9934 and Adj. R² value of 0.9836, the model explains the response data excellently and retains good predictive ability after adjusting for terms. The Pred. R² value of 0.7452 indicates reasonable accuracy in predicting new observations, while the C.V. of 7.07% underscores good precision and reliability of the experimental data. Moreover, the Adequate Precision value of 30.6285 (greater than 4) indicates the model’s utility in navigating the design space and making predictions within the studied mixture range. Y_IZD_ = *-65.1001 A + 16.1065 B + 3.04205 C + 128.852 AB + 149.826 AC + -30.2074 BC + -235.061 ABC + 154.06 AB(A-B) + 128.459 AC(A-C) + -32.2662 BC(B-C)* where A is oregano oil, B is Moringa, and C is cinnamon oil.

**Table 2:** Experimental design matrix with ANOVA for cubic model to relative contributions and potential interactions among the mixture components (oregano, moringa and cinnamon) influencing the inhibition zone diameter

Source	Sum of Squares	df	Mean Square	*F*-value	*P*-value	
Model	51.74	9	5.75	100.83	< 0.0001	significant
^(1)^Linear Mixture	10.47	2	5.23	91.79	< 0.0001	
AB	15.06	1	15.06	264.13	< 0.0001	
AC	9.85	1	9.85	172.68	< 0.0001	
BC	0.8661	1	0.8661	15.19	0.0080	
ABC	14.24	1	14.24	249.74	< 0.0001	
AB(A-B)	7.34	1	7.34	128.66	< 0.0001	
AC(A-C)	9.13	1	9.13	160.10	< 0.0001	
BC(B-C)	0.2263	1	0.2263	3.97	0.0934	
**Std. Dev.**	0.2388	**R²**		0.9934	**Std. Dev.**	0.2388
**Mean**	3.38	**Adjusted R²**		0.9836	**Mean**	3.38
**C.V. %**	7.07	**Predicted R²**		0.7452	**C.V. %**	7.07

Value of *P*-value less than 0.01 is significant

**Fig. 7:**
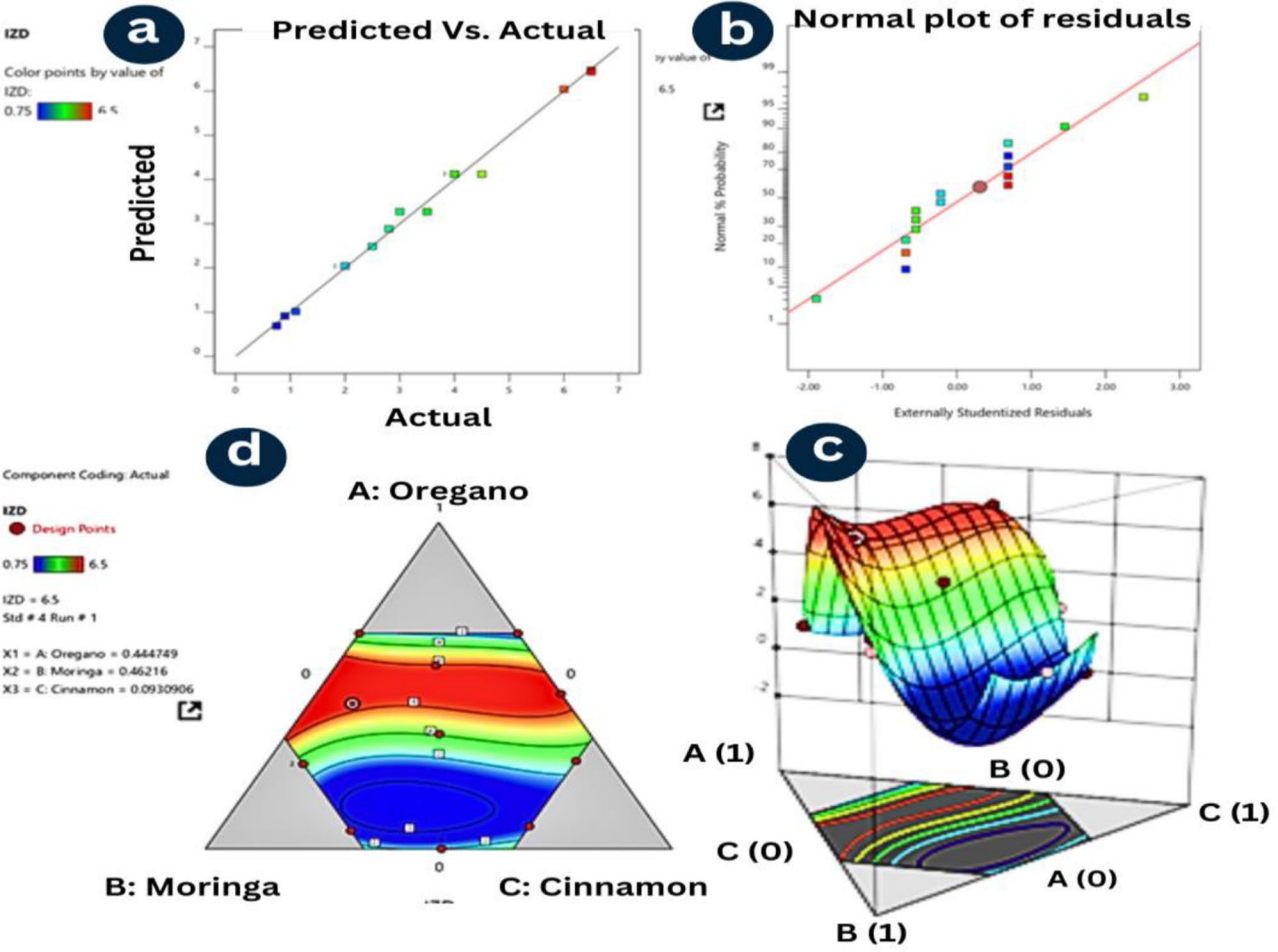
The optimal mixture composition that maximizes the inhibition zone diameter response, and the diagnostic plots indicate that the model assumptions are reasonably satisfied, lending confidence to the results obtained from the analysis of variance (ANOVA) test

As shown in Fig. 7, the normal plot of residuals shows that the residuals generally follow a straight line, indicating that the model’s assumptions of normality and constant variance are reasonably met. The 3D surface plot and the contour plot visually depict the response surface of IZD as a function of the three component proportions. The red and yellow regions indicate higher IZD values, while the blue regions represent lower values. The shape of the surface suggests that a combination of oregano and cinnamon, with a relatively lower proportion of moringa, tends to yield higher IZD values.

### MIC of the EOs mixture combination against all collected fungal strains

**Fig. 8:**
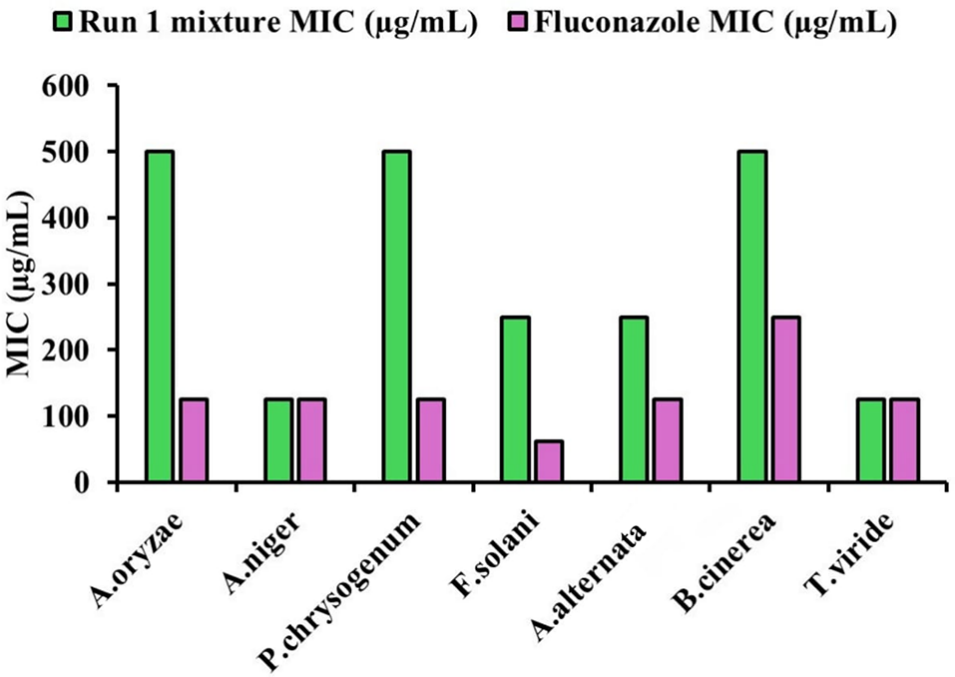
Minimal inhibitory concentration of the most effective mixture of EOs against the obtained fungal strains on PDA medium incubated at 25 °C for 3–5 days in comparison with a commercial standard antibiotic

The MIC values of the oils mixture and fluconazole against various fungal strains show some differences in effectiveness (Fig. 8). The oils mixture exhibited the highest MIC of 500 µg/mL against *P. chrysogenum*, *A. oryzae*, and *B. cinerea* while fluconazole was again more effective with an MIC of 125 µg/mL against *P. chrysogenum*, *A. oryzae*, with less effectiveness against *B. cinerea* (250 µg/mL). For *A. niger*, both the oils mixture and fluconazole had the same MIC of 125 µg/mL, suggesting equal efficacy. In the case of *F. solani*, the oils mixture and fluconazole had MICs of 250 µg/mL and 62.5 µg/mL, respectively, showing that fluconazole was more potent. Similarly, *A. alternata* had an MIC of 250 µg/mL for the oils mixture and 125 µg/mL for fluconazole, demonstrating fluconazole’s superior efficacy. Finally, *Trichoderma viride* had the lowest MIC for the oils mixture and fluconazole (125 µg/mL). Overall, fluconazole was generally more effective than the oils mixture against most fungal strains, except for *B. cinerea*, where the oils mixture showed a lower MIC.

### Gas chromatography for the three EOs

Based on the provided data in Table 3, the most abundant compounds in the different plant essential oils (EOs) are as follows: For Moringa, the most abundant compound was trans-13-Octadecenoic acid with a retention time of 29.57 and an area percentage of 22.62%. In Cinnamon, the predominant compound was (E)- Cinnamaldehyde with a retention time of 10.0979 and an area percentage of 24.4226%. For Oregano, the major compound is 2-methyl-5-(1-methylethyl) phenol (Carvacrol) with a retention time of 13.42 and an area percentage of 83.25%.

**Table 3:** Gac chromatography analysis for the most abundant compounds in oregano, moringa and cinnamon oils

	Retention Time (RT)	Compound Name	Area %	Molecular Formula
Moringa	25.62	Palmitic Acid methyl ester	3.98	C_17_H_34_O_2_
	26.39	n-Hexadecanoic acid	11.05	C_16_H_32_O_2_
	28.80	(E)-9-Octadecenoic acid, methyl ester	9.52	C_19_H_36_O_2_
	29.57	trans-13-Octadecenoic acid	22.62	C_18_H_34_O_2_
	44.30	Ethyl iso-allocholate	6.14	C_26_H_44_O_5_
Cinnamon	4.7443	Propylene Glycol	7.9458	C_3_H_8_O_2_
	8.0698	Linalool	1.0595	C_10_H_18_O
	10.0979	(E)-Cinnamaldehyde	24.4226	C_9_H_8_O
	10.7863	3-Allyl-6-methoxyphenol	18.5486	C_10_H_12_O_2_
	11.051	Methyl cis-cinnamate	4.4267	C_10_H_10_O_2_
Oregano	13.42	Cavarcol	83.25	C_10_H_14_O
	6.30	O-Cymene	6.34	C_10_H_14_O
	7.20	Ç-Terpinene	5.22	C_10_H_16_O
	13.18	Phenol, 5-Methyl-2-(1-Methylethyl)-	4.64	C_10_H_14_O
	5.34	Bicyclo [3.1.1]Heptane, 6,6-Dimethyl-2-Methylene-	0.54	C_10_H_16_O

### Toxicological potentials of EOs mixture against normal human skin fibroblast (HSF)

**Fig. 9:**
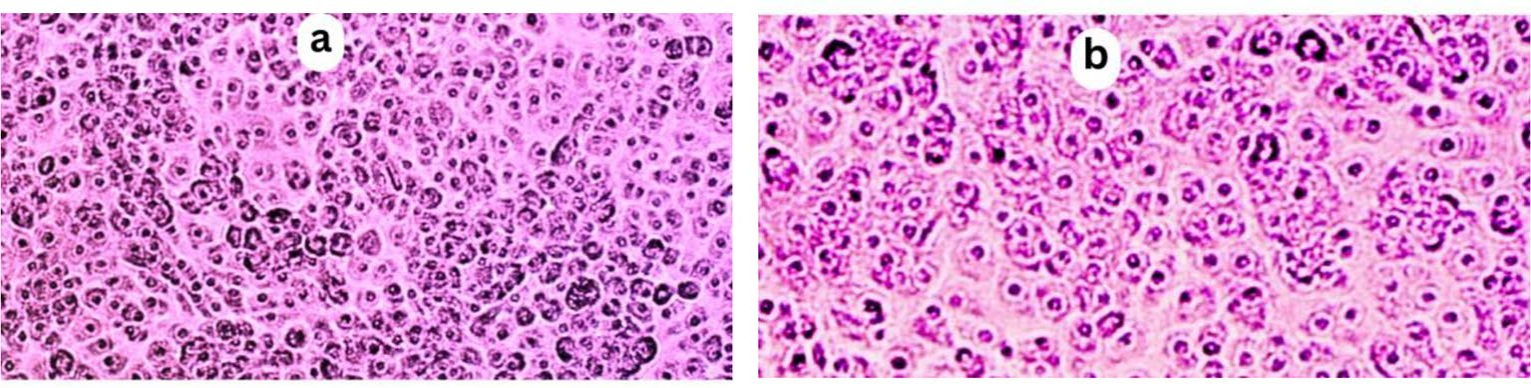
The cytotoxic effect of EOs against normal HSF. (**a**): Control treatment. (**b**): HSF cells treated with 100 µg/mL indicating normal and aggregated cells. Images were taken using a light microscope with magnification power of 200X

The cell viability assessment in Fig. 9 demonstrates a concentration-dependent effect on cell survival. At the lowest concentration of 0.01 µg/mL, cells thrive with a high survival rate of 98 ± 2.5%, nearly matching the blank control’s 100 ± 0.00%. As the concentration increases to 0.1 and 1 µg/mL, a slight decline in cell viability is noted at 93.60 ± 1.27%, and 91.49 ± 2.58% respectively. However, at higher concentrations of 10 and 100 µg/mL, a more substantial decrease in viability becomes evident at 88.65 ± 3.42%, and 87.64 ± 2.50% respectively, indicating escalated cytotoxic effects. The trend culminates at the highest concentration of 1000 µg/mL, where cell viability sharply plunges to 84.61 ± 0.72%, significantly lower than the control and lower concentrations, accentuating the increased cytotoxicity at this elevated level.

### Colorimetric measurements

**Fig. 10:**
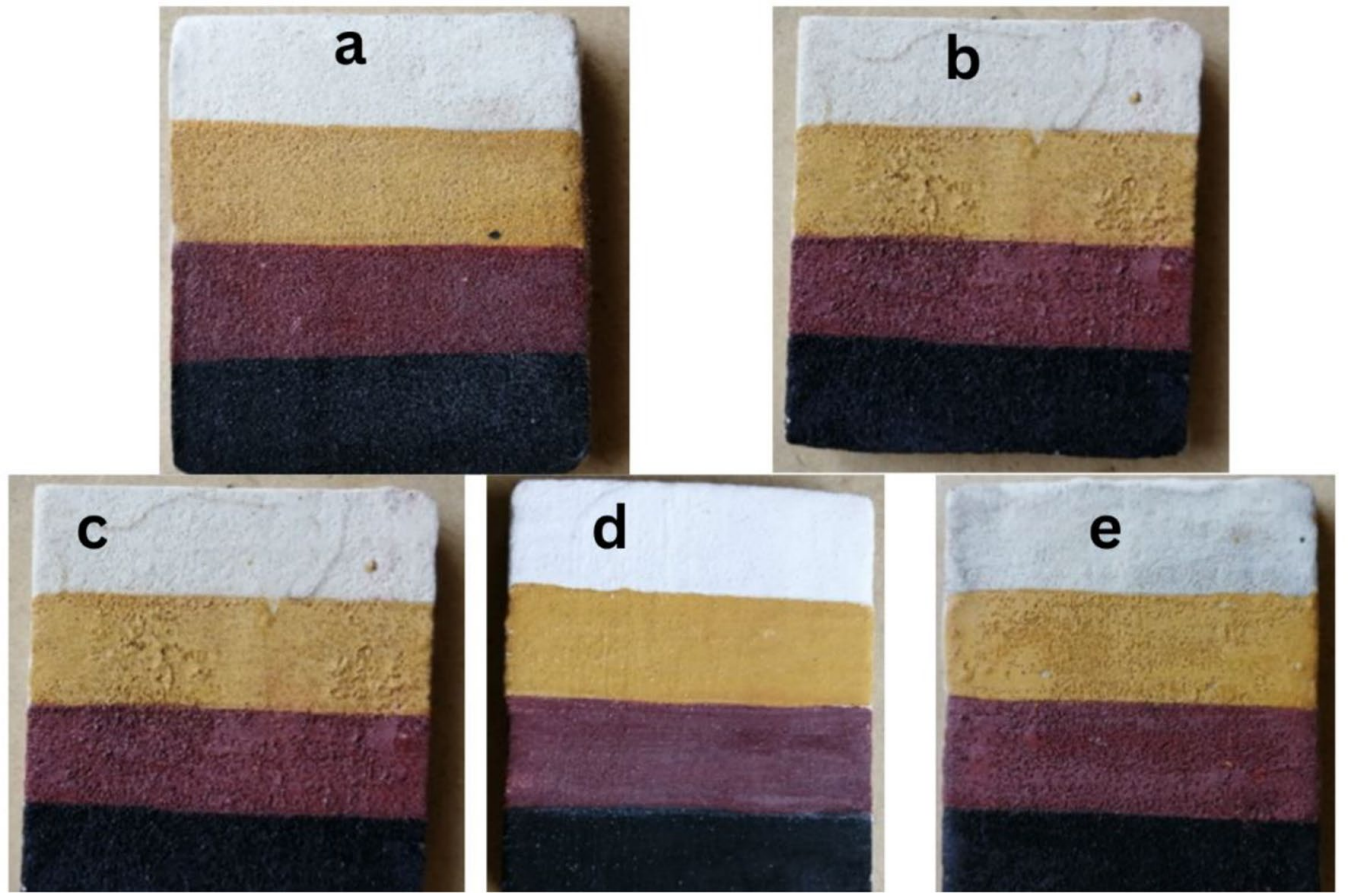
Comparative analysis of the effects of oregano, moringa, and cinnamon plant oils on different pigments using experimental painting samples. (**a**): Control. (**b**): Oregano. (**c**): Moringa. (**d**): Cinnamon, (**e**): Essential oils mixture

In a comparative analysis illustrated in Table 4; Fig. 10 of the effects of oregano, moringa, and cinnamon plant oils on different pigments, cinnamon exhibited the most pronounced impact on the white and red pigments, causing substantial alterations in colour characterized by significant changes in lightness and chromaticity values. Moringa, on the other hand, had the greatest effect on the yellow pigment, resulting in a substantial colour change marked by noticeable shifts in lightness and chromaticity. For the black pigment, both moringa and cinnamon caused comparable and significant colour alterations. Notably, oregano consistently showed the least impact across all pigments, inducing only minor colour changes. The effects of the three plant essential oils (Oregano, Moringa, and Cinnamon) on the colour values of white, red, black, and yellow pigments varied significantly. For the white pigment, Cinnamon caused the largest colour change (ΔE = 9.64), followed by Moringa (ΔE = 4.69), while Oregano had the least effect (ΔE = 1.73). With the yellow pigment, Moringa resulted in the most substantial alteration (ΔE = 16.31), Cinnamon caused a considerable shift (ΔE = 6.93), and Oregano had a minor impact (ΔE = 2.83). For the red pigment, Cinnamon (ΔE = 7.81) and Moringa (ΔE = 7.48) had comparable pronounced effects, while Oregano’s impact was relatively small (ΔE = 3.00). As for the black pigment, Moringa and Cinnamon exhibited similar noticeable colour changes (ΔE = 2.45 for both), with Oregano causing a slightly smaller alteration (ΔE = 1.41). Overall, Cinnamon and Moringa had the most significant effects across the pigments, particularly Cinnamon on the white pigment and Moringa on the yellow pigment, while Oregano consistently demonstrated the least impact. The results combining the effects of the three plant essential oils (Oregano, Moringa, Cinnamon) on the colour values of white pigment (Gypsum), red pigment (red ochre), black pigment (carbon black), and yellow pigments (yellow ochre).

**Table 4:** Comparative analysis of the effects of oregano, moringa and cinnamon essential oils on colour values of different pigments using experimental painting samples

Pigment	Treatment	L	a	b	ΔL	Δa	Δb	ΔE
White	Control	85	-2	14	-	-	-	-
	Oregano	84	-1	15	-1	1	1	1.73
	Moringa	82	1	16	-3	3	2	4.69
	Cinnamon	80	0	22	-5	2	8	9.64
Yellow	Control	36	4	44	-	-	-	-
	Oregano	34	6	44	-2	2	0	2.83
	Moringa	52	7	45	16	3	1	16.31
	Cinnamon	40	8	40	4	4	-4	6.93
Red	Control	31	18	17	-	-	-	-
	Oregano	29	17	15	-2	-1	-2	3.00
	Moringa	25	16	13	-6	-2	-4	7.48
	Cinnamon	25	14	14	-6	-4	-3	7.81
Black	Control	7	-1	0	-	-	-	-
	Oregano	6	0	0	-1	1	0	1.41
	Moringa	5	-2	1	-2	-1	1	2.45
	Cinnamon	5	0	1	-2	1	1	2.45

This table consolidates the L, a, b colour values, as well as the changes in L (ΔL), a (Δa), b (Δb), and the overall colour change (ΔE) for each pigment and treatment condition, allowing for easy comparison across the different pigments and essential oils.

## Discussion

The initial step in this study was the isolation and identification of various fungal genera from the collected samples. The results highlighted the dominance of *Aspergillus* sp. (50%) followed by *Penicillium sp*. and *Fusarium sp*. (16.7%). The presence of deteriorative fungi genera like *Alternaria* sp., *Trichoderma* sp, and *Botrytis* sp., although in lesser quantities, is noteworthy. These findings are consistent with previous studies that have reported the prevalence of these fungal genera in various environments and their potential for causing deterioration or spoilage [27, 28]. The phenotypic identification of the isolated fungi revealed diverse morphological features, such as septate hyphae, conidiophore structures, and spore arrangements, reflecting their taxonomic diversity and ecological roles. These observations align with the established morphological characteristics of these fungal genera.

Molecular identification using 18S rRNA gene analysis further corroborated the taxonomic affiliations of the isolated fungi and provided insights into their genetic relationships. The phylogenetic tree revealed varying degrees of genetic similarity among different isolates within the same genus, as well as between different genera. For instance, *Aspergillus niger* isolates exhibited close relatedness, while *Fusarium solani* isolates displayed genetic divergence within the species. These findings are consistent with previous studies that have utilized molecular techniques to delineate the genetic diversity and phylogenetic relationships among fungal isolates [5, 29, 30].

The minimum inhibitory concentration profile of the EOs mixture against various identified fungal strains presents compelling evidence of its efficacy. At concentrations ranging from 125 to 500 µg/ml, the EOs mixture exhibited complete inhibition against all tested fungal strains, including *Aspergillus oryzae*, *Aspergillus niger*, *Penicillium chrysogenum*, *Fusarium solani*, *Alternaria alternata*, *Botrytis cinerea*, and *Trichoderma viride.* This broad-spectrum antimicrobial activity is consistent with previous studies that have reported the antifungal properties of essential oils derived from oregano, moringa, and cinnamon. Interestingly, at the highest tested concentration of 1000 µg/ml, varying degrees of growth were observed for different fungal strains. This observation suggests a potential concentration-dependent effect, where lower concentrations may allow some fungal growth, while higher concentrations are more effective in inhibiting fungal growth. Similar concentration-dependent effects have been reported in other studies investigating the antfungal activity of plant essential oils [31, 32].

The exploration of antifungal activity of the EOs mixture against *Aspergillus niger* provides valuable insights into the potential interactions among the individual essential oil components. The diverse combinations of oregano, moringa, and cinnamon essential oils tested in the experimental runs revealed different antifungal effects, which could be attributed to the specific chemical compositions and mechanisms of action involved [33].

The ANOVA results for the cubic model reveal a highly significant model (*P*-value < 0.0001), indicating the ability of the model terms to explain the variation in the response variable (IZD) effectively. Significant model terms include linear mixture terms, interaction terms, and quadratic terms, suggesting the potential for antifungal interactions among the components. These findings are supported by previous studies that have reported antifungal activities among different plant essential oils and their constituents [5].

The optimal solution identified by the analysis suggests that a combination of oregano (44%), moringa (46%), and cinnamon (10%) yields the highest IZD value of approximately 6.5 cm. These findings are supported by previous studies that have reported the potent antimicrobial activities of oregano and cinnamon essential oils, attributed to their rich composition of phenolic compounds like carvacrol, thymol, and cinnamaldehyde [9, 34, 35]. The moderate contribution of moringa in the optimal mixture could be due to the antifungal interactions with the other components, as well as its own antifungal properties.

The GC analysis revealed the most abundant compounds present in each of the essential oil. These findings are consistent with previous reports on the chemical compositions of these essential oils and their potential bioactive compounds [36–38].

The colorimetric measurements provide insights into the effects of the individual essential oil on different pigments. The most pronounced impact was observed with cinnamon on the white and red pigments, and moringa on the yellow pigment, causing significant colour alterations. These findings suggest that the specific chemical compositions of these essential oils may interact with the pigments, leading to changes in their chromatic properties. Previous studies have reported the potential of plant essential oils to alter the colour and stability of pigments, which could be attributed to their antioxidant properties and interactions with pigment molecules [39, 40].

The results of this study highlight the promising antifungal potential of the EOs mixture against various deteriorative fungal strains. The antifungal interactions among the individual essential oil components, particularly oregano and cinnamon, contribute to enhance its activity. These findings have implications for the development of eco-friendly and sustainable strategies for preventing fungal deterioration in various settings, such as cultural heritage sites, food processing facilities, and agricultural applications. Future research could further explore the mechanisms of action underlying the antifungal activities of the EOs mixture, as well as investigate its potential applications in different contexts. Additionally, studying the effects of different extraction methods, environmental conditions, and storage conditions on the chemical compositions and bioactivities of the essential oils could provide valuable insights for optimizing their efficacy.

Furthermore, the observed impacts on pigment colour properties suggest potential applications of these essential oils in the field of dyes, pigments, and colorants, where their ability to alter chromatic properties could be leveraged for specific applications. However, further investigations are needed to understand the underlying mechanisms and potential implications of these colour alterations.

## Conclusion

The present study highlights the promising antifungal potential of a mixture of plant essential oils derived from oregano, moringa, and cinnamon against various deteriorative fungal strains. The systematic evaluation of the EOs mixture revealed its broad-spectrum antifungal activity, with complete inhibition observed at lower concentrations against *Aspergillus oryzae*, *Aspergillus niger*, *Penicillium chrysogenum*, *Fusarium solani*, *Alternaria alternata*, and *Botrytis cinerea.* Notably, the exploration of antifungal interactions among the individual essential oil components identified an optimal mixture composition comprising oregano, moringa, and cinnamon, exhibiting potent inhibitory effects against the fungal strain *Aspergillus niger*. The study also provided insights into the effects of these essential oils on pigment colour properties and their chemical compositions, suggesting potential applications in diverse fields such as cultural heritage preservation, food processing, and the development of eco-friendly antifungal agents. Furthermore, the observed antifungal activities pave the way for future research into the underlying mechanisms of action and the optimization of essential oil mixtures for targeted applications. Overall, the findings of this study contribute to the growing body of knowledge on the antifungal properties of plant essential oils and their potential as sustainable alternatives to conventional antifungal agents, while also highlighting their potential in other domains such as pigment and dye industries.

## Data Availability

The raw data and analyzed data used during the current study are available from the corresponding author upon reasonable request. *Penicillium chrysogenum* isolate QENA 2 was deposited in Genbank https://www.ncbi.nlm.nih.gov/nuccore/OQ726565.1. *Botrytis cinerea* isolate QENA 4 was deposited in Genbank. https://www.ncbi.nlm.nih.gov/nuccore/OQ726567.1. *Aspergillus oryzae* isolate QENA 5 was deposited in Genbank. https://www.ncbi.nlm.nih.gov/nuccore/OQ726568.1. *Aspergillus niger* isolate QENA 7 was deposited in Genbank. https://www.ncbi.nlm.nih.gov/nuccore/2473234566. *Alternaria alternata* isolate QENA 8 ws deposited in Genbank. https://www.ncbi.nlm.nih.gov/nuccore/OQ726571.1. *Fusarium solani* isolate QENA 10 was deposited in Genbank. https://www.ncbi.nlm.nih.gov/nuccore/OQ726572.1. *Trichoderma viride* isolate QENA 1 was deposited in Genbank. https://www.ncbi.nlm.nih.gov/nuccore/OQ726564.1
